# The Prognostic Significance of Whole Blood Global and Specific DNA Methylation Levels in Gastric Adenocarcinoma

**DOI:** 10.1371/journal.pone.0015585

**Published:** 2010-12-23

**Authors:** Mansour S. Al-Moundhri, Maryam Al-Nabhani, Letizia Tarantini, Andrea Baccarelli, Jennifer A. Rusiecki

**Affiliations:** 1 Medical Oncology Unit, Department of Medicine, College of Medicine and Health Sciences, Sultan Qaboos University (SQU), Muscat, Sultanate of Oman; 2 Department of Genetics, College of Medicine and Health Sciences, Sultan Qaboos University (SQU), Muscat, Sultanate of Oman; 3 Center of Molecular and Genetic Epidemiology, Department of Environmental and Occupational Health, Fondazione IRCCS Ca' Granda Ospedale Maggiore Policlinico and Università degli Studi di Milano, Milan, Italy; 4 Department of Preventive Medicine and Biometrics, Uniformed Services University, Bethesda, Maryland, United States of America; University of Barcelona, Spain

## Abstract

**Background:**

Epigenetics, particularly DNA methylation, has recently been elucidated as important in gastric cancer (GC) initiation and progression. We investigated the clinical and prognostic importance of whole blood global and site-specific DNA methylation in GC.

**Methods:**

Genomic DNA was extracted from the peripheral blood of 105 Omani GC patients at diagnosis. DNA methylation was quantified by pyrosequencing of global DNA and specific gene promoter regions at 5 CpG sites for CDH1, 7 CpG sites for p16, 4 CpG sites for p53, and 3 CpG sites for RUNX3. DNA methylation levels in patients were categorized into low, medium, and high tertiles. Associations between methylation level category and clinicopathological features were evaluated using χ^2^ tests. Survival analyses were carried out using the Kaplan-Meier method and log rank test. A backward conditional Cox proportional hazards regression model was used to identify independent predictors of survival.

**Results:**

Older GC patients had increased methylation levels at specific CpG sites within the CDH1, p53, and RUNX-3 promoters. Male gender was significantly associated with reduced global and increased site-specific DNA methylation levels in CDH1, p16, and p53 promoters. Global DNA low methylation level was associated with better survival on univariate analysis. Patients with high and medium methylation vs. low methylation levels across p16 promoter CpG sites, site 2 in particular, had better survival. Multivariate analysis showed that global DNA hypermethylation was a significant independent predictor of worse survival (hazard ratio (HR) = 2.0, 95% CI: 1.1–3.8; *p* = 0.02) and high methylation mean values across p16 promoter sites 1–7 were associated with better survival with HR of 0.3 (95% CI, 0.1–0.8; *p* = 0.02) respectively.

**Conclusions:**

Analysis of global and site-specific DNA methylation in peripheral blood by pyrosequencing provides quantitative DNA methylation values that may serve as important prognostic indicators.

## Introduction

Gastric cancer (GC) is a common malignancy that is a leading cause of cancer mortality worldwide [1]. GC has been linked to Helicobacter infection and environmental exposures including: smoking, salted fish, and low intake of fruit and vegetables [2], [3], . While these exposures are very common, very few exposed individuals develop GC. Therefore, it has been postulated that genetic factors such as single nucleotide polymorphisms in genes in several cellular pathways may increase GC risk [2], [3], [4], . In addition, studies have recently begun to elucidate the role of epigenetics, in particular DNA methylation, in GC initiation and progression [9], [10], [11]. Global DNA hypomethylation is associated with genomic instability, while DNA hypermethylation at CpG islands in or near gene promoter regions is associated with gene “silencing” [10], [12], [13]. Global genomic DNA methylation in cancerous gastric tissues has been found to be significantly lower than in non-cancerous tissues and shows a gradual increase in hypomethylation from normal gastric mucosa to chronic atrophic gastritis, severe, and intestinal metaplasia [10], [12], [13]. Global DNA hypomethylation occurs at an early stage in gastric carcinogenesis and may therefore serve as a novel biomarker of gastric neoplasia [12]. In contrast, several genes have been found to exhibit promoter hypermethylation resulting in gene silencing in GC. It has been suggested that the hypermethylation of the tumor suppressor genes, RUNX3 and TSLC1, may have value as molecular diagnostic markers, and hMLH1 and p16 methylation may predict stomach cancer risk [14]. CDH1 promoter hypermethylation frequently occurs in gastric carcinomas with a diffuse histotype and is significantly associated with down-regulated E-cadherin expression [15].

The potential diagnostic and prognostic value of promoter hypermethylation in the tissue and serum of patients with GC has been shown, particularly for the promoters of the p16, CDH1, GSTP1, and APC genes [16], [17]. More recently, the use of non-target tissue such as whole blood has been suggested as a useful biomarker in cancers such as gastric, lung, breast, bladder, and head and neck cancers [18], [19], [20], [21], [22]. Hou et al demonstrated that LINE-1 hypomethylation increased gastric cancer risk [OR  = 1.4 (95% CI  =  (0.9–2.0)] [18]. Hsiung et al found that hypomethylation LRE1 sequence resulted in a significant increase risk for head and neck cancer in a case-control study[19]. Moreover, in another case-control study, there was an association between leukocyte DNA hypomethylation with increased risk of developing bladder cancer, independent of smoking and other assessed risk factors[21]. Global DNA hypomethylation and locus-specific methylation patterns in peripheral blood DNA were found to be a potential surrogate markers for breast cancer risk [20], [22]. Therefore, with above data suggesting usefulness of analysis of global and specific methylation and cancer risk predisposition coupled with prognostic data in target tissue and serum, we studied the prognostic significance of whole blood DNA methylation levels both globally (estimated in LINE-1 repeated elements) and in the promoter regions of the p16, CDH1, p53, and RUNX3 genes using pyrosequencing in an Omani GC population.

## Materials and Methods

### Study participants

The study population consisted of a series of unrelated GC patients who were diagnosed between 2004–2008 at two main hospitals in the Sultanate of Oman (Sultan Qaboos University Hospital and Royal Hospital). The Medical Research and Ethics Committee of the College of Medicine of Sultan Qaboos University and the Institutional Review Board of the Uniformed Services University approved the study design. Participants of this study as part of epigenetics of gastric cancer in Oman project were provided with informed written consent prior to study participation in compliance with the Declaration of Helsinki.

### Blood Collection and DNA extraction

Ten milliliters of blood was collected from each participant in an EDTA tube and stored frozen until DNA extraction at time of diagnosis or referral to the treating center for consideration for chemotherapy or chemo-radiotherapy. Whole blood DNA extraction was performed using a commercial kit (Gentra Puregene DNA Purification kit, Qiagen, Gaithersburg, MD, USA) and stored until processing for analysis.

#### Bisulfite treatment

Five hundred nanograms of genomic DNA was treated using the EZ DNA Methylation-Gold Kit (D5007, Zymo Research, Orange, CA, USA) according to the manufacturer's instructions. The bisulfite-treated DNA was eluted with 30 µl MElution buffer. Several methods exist to measure DNA methylation. Recently, a new bisulfite-based PCR method was developed to assess global DNA methylation using amplified repetitive LINE-1 elements that are normally heavily methylated [23]. Since it is estimated that more than one-third of DNA methylation occurs in repetitive elements, analyzing repetitive element methylation can serve as a surrogate marker for global genomic DNA methylation [23]. The same method can be used to measure CpG island methylation in gene promoter regions.

#### PCR and Pyrosequencing

We performed 50 µl PCR reactions using GoTaq Green Master mix (M7123, Promega, Madison, WI, USA), 10 pmol each of forward and reverse primers, and 50 ng bisulfite-treated genomic DNA (the primer sequences and PCR conditions are shown in Table 1. The gene-specific assays allowed for the analysis of multiple adjacent CpG sites within the promoter regions of each gene. We measured CpG methylation in genes at 5 sites in CDH1, 7 sites in p16, 4 sites in p53, and 3 sites in RUNX3. In our statistical analysis, we considered both site-specific and mean CpG methylation across sites for each gene. The LINE-1 assay measured DNA methylation at three adjacent CpG sites. Because the value of measuring methylation at a single CpG site within repeated sequences is undetermined, our statistical analysis considered only the average methylation in the three LINE-1 CpG sites. DNA methylation level was quantified using bisulfite-PCR and pyrosequencing. Briefly, a biotin-labeled primer was used to purify the final PCR product using Sepharose beads. The PCR product was bound to Streptavidin Sepharose High Performance (AmershamBiosciences, Uppsala, Sweden), purified, washed, denatured with 0.2 mol/L NaOH, and washed again using the Pyrosequencing Vacuum Prep Tool (Pyrosequencing, Inc., Westborough, MA) according to the manufacturer's instructions. Pyrosequencing primer (0.3 µM) was then annealed to the purified single-stranded PCR product and pyrosequencing was performed using the PyroMark Q96MD pyrosequencing system (Qiagen, Inc, Hilden, Germany).

**Table 1:** Primer sequences and PCR conditions for global DNA methylation and specific gene methylation.

Sequence ID		Forward primer (5′ to 3′)	Reverse primer (5′ to 3′)	Sequencing primer (5′ to 3′) PCR	PCR conditions	No. CpG sites analyzed
Global DNA Methylation	LINE-1	TTTTGAGTTAGGTGTGGGATATA	BIOTIN-AAAATCAAAAAATTCCCTTTC	AGTTAGGTGTGGGATATAGT	95°C for 5 min (1 cycle), 95°C for 30 s, 50°C for 30 s, 72°C for 30 s (45 cycles), 72°C for 5 min(1cycles), 6°C for ∞	3
Gene-Specific Methylation	P53	P53BIOTIN-TTAGGAGTTTATTTAATTTAGGGAAG	TATCCAACTTTATACCAAAAACCTC	TCCAAAAAACAAATAACTACTAAACTC	95°C for 5 min (1 cycle), 95°C for 1 min, 57°C for 1 min, 72°C for 1 min (50cycle), 72°C for 5 min (1 cycle), 4°C for ∞	4
Gene-Specific Methylation	CDH1	TTTGATTTTAGGTTTTAGTGAGT	BIOTIN-ACCACAACCAATCAACAA	TAGTAATTTTAGGTTAGAGG	95°C for 5 min (1 cycle), 95°C for 30 s, 55°C for 30 s, 72°C for 30 s (40 cycle), 72°C for 5 min (1 cycle), 6°C for ∞	5
Gene-Specific Methylation	P16	AGGGGTTGGTTGGTTATTAG	BIOTIN-CTACCTACTCTCCCCCTCTC	GGTTGGTTATTAGAGGGT	95°C for 5 min (1 cycle), 95°C for 30 s, 58°C for 40 s, 72°C for 30 s (45 cycle), 72°C for 5 min (1 cycle), 6°C for ∞	7
Gene-Specific Methylation	RUNX3	GGGTATTTTTTATTTTTATTGTT	BIOTIN-ACAACCCCAACTTCCTCTA	GTATTTATTTTGAAGG	95°C for 5 min (1 cycle), 95°C for 1 min, 52°C for 30 s, 72°C for 30 s (50 cycle), 72°C for 5 min (1 cycle), 4°C for ∞	3

The cut-off values for discrimination of methylation levels for global and specific whole blood DNA methylation were categorized based as follows: low (<33 percentile), medium (≥33 and <66 percentile), and high (≥66% percentile) (Table S1). In the text, the terms “hypomethylation” and “hypermethylation” are used interchangeably with words “low” and “high” methylation respectively.

### Statistical analysis

Associations between methylation level category and clinicopathological features were evaluated using χ^2^ tests. Survival time was defined as the interval between a biopsy-proven diagnosis and death or the last known follow-up examination, whichever came first. The date of death was obtained from medical records or telephone contact. The Kaplan-Meier method was used to estimate overall survival time, and statistical significance was determined using the log-rank test. A backward conditional Cox proportional hazards regression model was used for multivariate analyses that included age (<50 years and ≥50 years), gender, tumor depth of invasion (T1 and T 2 vs. T3 and T4), presence or absence of lymph node metastases, overall stage (I and II vs. III and IV), tumor differentiation (well vs. moderate and poor), use of either chemotherapy or chemo-radiotherapy, and methylation variables that showed a statistically significant association with survival from univariate analysis. *P*-values less than 0.05 were considered statistically significant. All data analyses were performed using SPSS 16.0 software (SPSS, Chicago, IL, USA).

## Results

One hundred and five GC patients that were diagnosed during 2004–2008 were included. The age range for the GC patients was 19–83 years. The means and standard deviations of patient was 56.2±12.2. Sixty percent of participants were male and forty percent were female.

### Global and specific DNA methylation levels in association with clinicopathological features

Using χ^2^ tests we examined the relationship between global and specific DNA methylation levels age, gender and the following cancer prognostic factors: T stage, lymph node involvement, overall stage, and tumor differentiation (Table S1).

Older age (≥50years) was associated with CDH1 promoter hypermethylation at sites 3, 5, and on average across the five CDH1sites, p53 promoter hypermethylation at site 3 and on average across the four p53 sites, and RUNX3 promoter hypermethylation at site 1.

Male gender was associated with Global DNA hypomethylation, CDH1 promoter hypermethylation at sites 2,3, 4,5, and on average across the five CDH1sites, p16 promoter hypermethylation at sites 2 and 5 and hypomethylation at sites 3 and 6, p53 promoter hypermethylation at sites 1-4 and on average across the four p53 sites.

In terms of tumor characteristics, advance T stage was associated with RUNX3 promoter hypomethylation at site 3. Lymph node involvement was associated with p53hypomethylation at sites 1, 3, 4 and on average across the four sites, and RUNX3 promoter hypomethylation at site 2. Advanced overall stage was associated with p16 promoter hypermethylation at site 5 and hypomethylation at p53 site 1.

We found no association between smoking status and methylation patterns in the 16 patients who were confirmed to be smokers (data not shown). The serology for Helicobacter pylori (HP) infection was available for 41 patients. There were no significant associations between HP infection and DNA methylation patterns.

### Survival analysis

**Figure 1:**
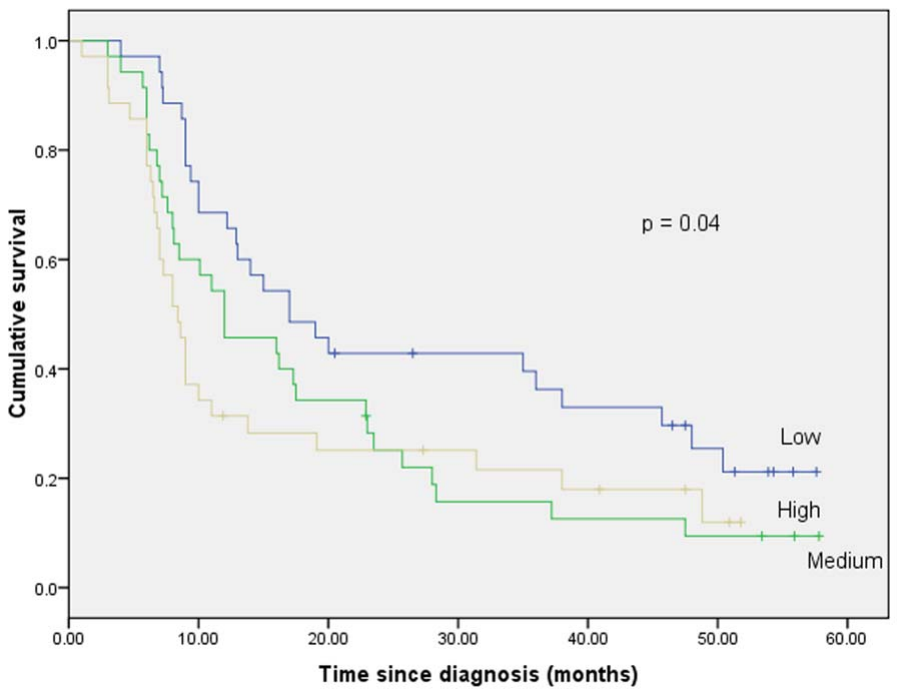
Cumulative survival of GC patients according to whole global (Line-1) blood DNA methylation values.

At the time of analysis (June 2010), the median survival times for patients classified as having low, medium, and high global DNA methylation levels were: 17.0 months (95% CI: 10.0–24.0), 12.0 months (95% CI: 5.2–18.8), and 8.4 months (95% CI: 7.0–9.8) respectively. These results suggest that patients with hypomethylated compared to those with medium or hypermethylated blood global DNA values have better survival (*p* = 0.04) (Figure 1).

**Figure 2:**
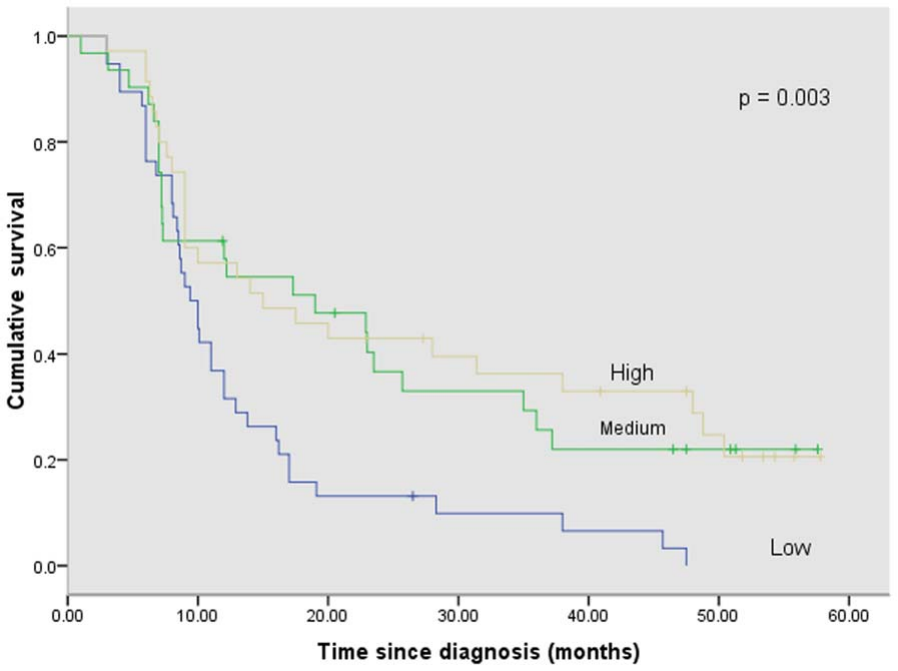
Cumulative survival of GC patients according to P16 (mean value of 7 sites) whole blood DNA methylation values.

**Figure 3:**
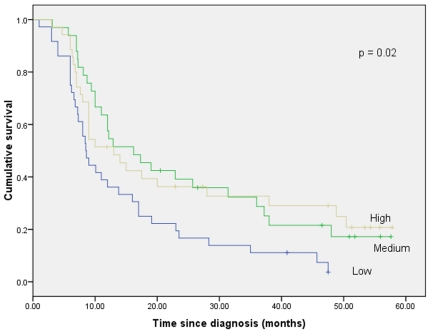
Cumulative survival of GC patients according to P16 (site 1) whole blood DNA methylation values.

We found that patients classified as having hypomethylation in p16 based on the mean methylation value across sites and in particular at site 2 had worse survival than patients with medium or high methylation levels (Figure 2 and 3). The median survival times for patients classified as having low, medium, and high p16 DNA methylation levels (the mean across all seven p16 sites) were 9.4 months (95% CI: 7.4–11.4), 19.0 months (95% CI: 5.0–33.0), and 15 months (95% CI: 3.4–26.6) respectively, (*p* = 0.003) (Figure 2). The median survival times for patients with low, medium, and high DNA methylation at site 2 of the p16 gene were 8.5 months (95% CI: 7.0–10.0), 16.2 months (95% CI: 8.3–24.1), and 14 months (95% CI: 0.1–15.6) respectively, (*p* = 0.02) (Figure 3).

Multivariate analysis showed that a hypermethylated global DNA value at diagnosis was a significant independent predictor of worse survival with a HR of 2.0 (95% CI, 1.1–3.8; *p* = 0.02) (Table 2). In contrast, medium and high methylation based on the mean methylation at sites 1–7 of the p16 gene were associated with better survival with HRs of 0.5 (95% CI, 0.2–0.7; *p* = 0.05) and 0.3 (95% CI, 0.1–0.8; *p* = 0.02) respectively. Other loci were not found to be significant independent predictors of survival.

**Table 2:** Multivariate survival analysis of prognostic factors in GC patients.

		*HR*	±*95% CI*	*p-value*
**Sex**	Male	1	reference	
	Female	0.9	0.5–1.5	0.6
**Age**	<50 years	1	reference	
	≥50 years	1.3	0.8–2.2	0.3
**T stage**	T1 and T2	1	reference	
	T3 and T4	0.7	0.3–1.6	0.5
**Lymph node involvement**	Negative	1	reference	
	Positive	1.8	0.7–4.6	0.2
**Overall TNM stage**	Stage 1 and II	1	reference	
	Stage III and IV	2.0	0.7–5.5	0.2
**Histology grade**	G1	1	reference	
	G2	0.2	0.1–1.7	0.4
	G3	0.3	0.1–1.8	0.5
**Chemotherapy/Chemoradiotherapy**	No	1	reference	
	Yes	0.4	0.3–0.7	0.0001
**Global DNA (Line-1)**	Low	1	reference	
	Medium	1.7	0.9–3.0	0.08
	High	2.0	1.1–3.8	0.02
**P16 (mean value of 7 sites)**	Low	1	reference	
	Medium	0.5	0.3–1.0	0.05
	High	0.3	0.1–0.8	0.02
**P16 (site 1)**	Low	1	reference	
	Medium	0.9	0.5–1.7	0.7
	High	1.4	0.5–4.0	0.5

HR indicates hazard ratio; CI, confidence interval.

## Discussion

Few studies have addressed the prognostic value of epigenetic alterations using pyrosequencing of DNA derived from the whole blood of patients with solid malignancies [19], [24]. To address this gap in knowledge, we used pyrosequencing to examine whether whole blood DNA methylation level, both globally (Line-1) and in the promoter regions of the p16, CDH1, p53, and RUNX3 genes was associated with prognosis.

Several important observations were made with regard to methylation patterns and clinicopathological characteristics. Our data suggest that older GC patients have increased whole blood DNA methylation levels in the promoters of the CDH1, p53, and RUNX3 genes. Although, this observation has not been reported before in GC, it is consistent with other studies that have reported increased methylation levels in other specific genes such as RASSF1A and hMLH1 in several of cancers including GC [24], [25], [26]. moreover, Hypermethylation of tumor suppressor genes, including p16, has been found in several aging tissues [27]. Gene-specific hypermethylation may be related to increased DNA damage and increased duration of carcinogen exposure with aging and could predispose to carcinogenesis.

We observed a significant association between gender and DNA methylation, with men being more likely than women to have high whole blood DNA methylation levels in the promoters of the CDH1, p16, and p53 genes. In contrast, males were more likely to have reduced global DNA methylation levels. These findings raise the issue of the biological influence of gender on carcinogenesis. Several studies suggest that GC growth and migration is modulated by sex steroid hormones, which is similar to findings in lung and bladder cancer [19], [28].

No previous studies have addressed global and specific DNA methylation levels in whole blood in association with G.C. cancer survival. On the other hand, few studies demonstrated the association between global DNA hypomethylation in target cancerous or non-target tissues such serum or whole blood and clinical outcome in other cancers. It was demonstrated that a high serum LINE-1 hypomethylation level was an independent predictor of shortened overall survival in 85 patients with hepatocellualr cancer [29]. Another study included 59 patients with microdissected ovarian cancer tissue showed that excessive LINE-1 hypomethylation was associated with a shortened overall survival [30]. In a large prospective cohort study of health care professionals, it was shown that global DNA hypomethylation as measured in LINE-1 is independently associated with poor survival among patients with colon cancer [31]. In contrast, we found that patients with low vs. medium or high whole blood global DNA values had better survival as indicated by the multivariate analysis showing that hypomethylation is an independent prognostic factor as shown in Figure 1 and Table 2. The reasons behind this unexpected observation is unclear, however, several postulates can be made. The use of non-target tissue such as whole blood DNA methylation (global and specific) in GC patients that can be modulated by various environmental factors and dietary deficiencies (such folate deficiency) [19]. Therefore, for whole blood DNA methylation to serve as a biomarker for GC, it should ideally be correlated with tissue methylation patterns. Two recent publications in colon cancer showed that there was a positive relationship between methylation in leukocytes and colonic tissue in colorectal tumors - albeit inability to distinguish between disease groups in one of these studies- suggesting potential usefulness because of ease of accessibility [32]–[33]. Furthermore, the use of pyrosequencing as an accurate and quantitative analysis is different from qualitative techniques used in many of the other studies.

We found that the patients classified as having high or medium according to the mean value of all studied p16 promoter CpG sites (1–7), and in particular at site 2, had better survival than those classified as having p16 low values and shown in Figure 2 and 3. In multivariate analysis, overall high methylation value at sites 1–7 in the p16 gene, in particular, was found to have independent prognostic significance as shown in Table 2. There were no previous studies that examined the prognostic significance of whole blood DNA in GC. Therefore, with the paucity of studies, the relationship between methylation status and prognosis remains a controversial area even when studied in gastric cancer tissue. Kissa et al demonstrated that RASSFIA, APC, and RAR-β2 promoter hypermethylation were significantly correlated with improved survival in GC patients [34]. Moreover, An et al showed that concordant hypermethylation of multiple genes (p16, hMLH1, MINT1, MINT2, MINT25, and MINT31) was associated with better survival [35]. It has been postulated that GC tumors with epigenetic alterations are less aggressive and patients with these types of tumors have improved prognosis compared to those with tumors that harbor other genetic alterations [34]. Furthermore, it has been shown in head and neck cancer that patients with CDH1 hypermethylation have significantly better overall survival than those without hypermethylation [36]. Marsit et al postulated that inactivation of the CDH1 gene by hypermethylation may lead to a less biologically aggressive tumor phenotype and greater sensitivity to treatment thereby providing a survival advantage [36]. In contrast Zazula et al and Graziano et al showed that CDH1 promoter hypermethylation was associated with worse prognosis [15], [37]. Studies have shown that hypermethylation of the p16 promoter results in worse survival in high-intermediate-risk and high-risk diffuse large B cell lymphoma and colorectal cancer patients [38], [39].

The correlation between site-specific DNA methylation levels and poor prognostic features such as the depth of tumor invasion and lymph node metastasis is of particular interest given the survival patterns described above and the role of apoptotic and adhesion regulating genes play in tumor growth and invasion. We found that hypomethylation of several specific gene promoter sites in whole blood DNA were associated with poor prognostic features. In particular, hypomethylation of p53 at sites 1, 3, 4, and overall, and of RUNX3 at site 2 was associated with lymph node involvement. Advanced T stage presentation was associated with hypomethyaltion of RUNX3 at site 3. Although, promoter region hypermethylation is the prime mechanism of transcriptional silencing of various tumor suppressors involved in carcinogenesis, it is increasingly recognized that promoter region hypomethylation also alter the transcriptional activation of different genes including MAGE, S100A4, and synuclein γ [40], [41], [42]. Moreover, Sato et al demonstrated that gene hypomethylation associated with overexpression of multiple genes that contributes to carcinogenesis in pancreatic adenocarcinoma [43]. Lin et al suggested that of the extent of hypomethylation correlates with poor prognostic features in some cancers[29]. Taken together, the current study suggests that promoter-specific low methylation levels results in worse survival in some genes such as p16 with correlation- in other genes- with poor prognostic features possibly by altering affected gene expression. However, it should be highlighted that our understanding of the role of specific promoter hypomethylation in carcinogenesis and prognosis is limited, particularly in whole blood.

In conclusion, our preliminary analysis suggests that epigenetic changes detected in whole blood DNA are associated with several prognostic factors and therefore these results may benefit GC patients in terms of treatment protocol design and follow-up. Moreover, the current study demonstrates the feasibility of pyrosequencing for quantifying DNA methylation and may thus serve as a non-invasive prognostic tool.

## Supporting Information

Table S1 The association between global DNA methylation and specific gene methylation and clinicoplatological features.
(DOC)
