# Supplementary material for: The Prognostic Significance of Whole Blood Global and Specific DNA Methylation Levels in Gastric Adenocarcinoma
Source: PLoS One. 2010 Dec 23;5(12):e15585. doi: 10.1371/journal.pone.0015585 (PMC3009731; doi:10.1371/journal.pone.0015585)
Supplement: Table S1 — The association between global DNA methylation and specific gene methylation and clinicoplatological features. (DOC) [file pone.0015585.s001.doc]

**The association between global DNA methylation and specific gene methylation# and clinicoplatological features**

| **Locus** | **Age** | |  | **Gender** | |  | **Histological grade** | | |  | **T stage** | |  | **Lymph node** | |  | **TNM stage** | |  |
| --- | --- | --- | --- | --- | --- | --- | --- | --- | --- | --- | --- | --- | --- | --- | --- | --- | --- | --- | --- |
|  | **<50** | **≥50** | ***p*•** | **M** | **F** | ***p*** | **G1** | **G2** | **G3** | ***p*** | **T1/2** | **T3/4** | ***p*** | **–Ve** | **+Ve** | ***p*** | **I/II** | **III/Iv** | ***p*** |
| **(Line 1): Global** Mean (3 sites) |  |  | 0.7 |  |  | 0.0001 |  |  |  | 0.3 |  |  | 0.2 |  |  | 0. 4 |  |  | 0.9 |
| Low* (<76.67) | 10 | 25 |  | 30 | 05 |  | 00 | 17 | 18 |  | 08 | 27 |  | 04 | 31 |  | 4 | 31 |  |
| Medium (76.67-77.64) | 7 | 28 |  | 20 | 15 |  | 01 | 14 | 20 |  | 03 | 32 |  | 07 | 28 |  | 4 | 31 |  |
| High (>77.64) | 9 | 26 |  | 13 | 22 |  | 02 | 10 | 23 |  | 05 | 30 |  | 03 | 32 |  | 3 | 32 |  |
| **CDH-1** |  |  |  |  |  |  |  |  |  |  |  |  |  |  |  |  |  |  |  |
| **site 1** |  |  | 0.2 |  |  | 0.9 |  |  |  | 0.9 |  |  | 0.8 |  |  | 0.7 |  |  | 0.5 |
| Low (<5.48) | 10 | 23 |  | 20 | 13 |  | 01 | 11 | 21 |  | 06 | 27 |  | 03 | 30 |  | 05 | 28 |  |
| Medium (5.48-6.69) | 9 | 23 |  | 20 | 12 |  | 01 | 12 | 19 |  | 04 | 28 |  | 02 | 30 |  | 02 | 30 |  |
| High (>6.69) | 4 | 27 |  | 19 | 12 |  | 01 | 11 | 19 |  | 05 | 26 |  | 04 | 27 |  | 04 | 27 |  |
| **site 2** |  |  | 0.06 |  |  | 0.001 |  |  |  | 0.4 |  |  | 0.9 |  |  | 0.7 |  |  | 0.9 |
| Low (<10.30) | 12 | 20 |  | 11 | 21 |  | 02 | 10 | 20 |  | 03 | 29 |  | 02 | 30 |  | 03 | 29 |  |
| Medium (10.30-12.44) | 7 | 25 |  | 24 | 08 |  | 01 | 14 | 17 |  | 04 | 28 |  | 03 | 29 |  | 04 | 28 |  |
| High(>12.44) | 4 | 28 |  | 24 | 08 |  | 00 | 10 | 22 |  | 04 | 28 |  | 04 | 28 |  | 04 | 28 |  |
| **site 3** |  |  | 0.02 |  |  | 0.001 |  |  |  | 0.4 |  |  | 0.4 |  |  | 0.7 |  |  | 0.5 |
| Low (<12.10) | 13 | 19 |  | 12 | 20 |  | 01 | 13 | 18 |  | 07 | 25 |  | 02 | 30 |  | 04 | 28 |  |
| Medium (12.10-16.17) | 6 | 26 |  | 21 | 11 |  | 02 | 09 | 21 |  | 03 | 29 |  | 03 | 29 |  | 02 | 30 |  |
| High (>16.17) | 4 | 28 |  | 26 | 06 |  | 00 | 12 | 20 |  | 05 | 27 |  | 04 | 28 |  | 05 | 27 |  |
| **site 4** |  |  | 0.3 |  |  | 0.01 |  |  |  | 0.3 |  |  | 0.5 |  |  | 0.3 |  |  | 0.9 |
| Low (<10.16) | 10 | 22 |  | 13 | 19 |  | 02 | 13 | 17 |  | 06 | 26 |  | 04 | 28 |  | 04 | 28 |  |
| Medium (10.16-12.67) | 8 | 24 |  | 23 | 09 |  | 00 | 13 | 19 |  | 06 | 26 |  | 01 | 31 |  | 04 | 28 |  |
| High (>12.67) | 5 | 27 |  | 23 | 09 |  | 01 | 8 | 23 |  | 03 | 29 |  | 04 | 28 |  | 03 | 29 |  |
| **site 5** |  |  | 0.01 |  |  | 0.007 |  |  |  | 0.5 |  |  | 0.9 |  |  | 0.5 |  |  | 0.2 |
| Low (<12.62) | 10 | 17 |  | 10 | 17 |  | 1 | 8 | 18 |  | 05 | 22 |  | 02 | 25 |  | 01 | 26 |  |
| Medium (12.62-14.70) | 10 | 28 |  | 26 | 12 |  | 2 | 13 | 23 |  | 06 | 32 |  | 02 | 36 |  | 05 | 33 |  |
| High (>14.70) | 1 | 21 |  | 17 | 05 |  | 0 | 9 | 13 |  | 03 | 19 |  | 03 | 19 |  | 04 | 18 |  |
| **Mean (5 sites)** |  |  | 0.02 |  |  | 0.001 |  |  |  | 0.5 |  |  | 0.4 |  |  | 0.2 |  |  | 0.9 |
| Low (<10.52) | 13 | 19 |  | 11 | 21 |  | 1 | 11 | 20 |  | 07 | 25 |  | 01 | 31 |  | 03 | 29 |  |
| Medium (10.52-12.45) | 6 | 26 |  | 23 | 09 |  | 2 | 12 | 18 |  | 03 | 29 |  | 05 | 27 |  | 04 | 28 |  |
| High (>12.45) | 4 | 28 |  | 25 | 07 |  | 0 | 11 | 21 |  | 05 | 27 |  | 03 | 29 |  | 4 | 28 |  |
| **p-16** |  |  |  |  |  |  |  |  |  |  |  |  |  |  |  |  |  |  |  |
| **site 1** |  |  | 0.6 |  |  | 0.2 |  |  |  | 0.2 |  |  | 0.2 |  |  | 0.05 |  |  | 0.5 |
| Low (<1.93) | 8 | 28 |  | 25 | 11 |  | 02 | 16 | 18 |  | 07 | 29 |  | 01 | 35 |  | 05 | 31 |  |
| Medium (1.93-2.59) | 7 | 26 |  | 16 | 17 |  | 01 | 9 | 23 |  | 02 | 31 |  | 05 | 28 |  | 03 | 30 |  |
| High (>2.59) | 11 | 24 |  | 21 | 14 |  | 00 | 15 | 20 |  | 07 | 28 |  | 07 | 28 |  | 02 | 33 |  |
| **site 2** |  |  | 0.8 |  |  | 0.0001 |  |  |  | 0.08 |  |  | 0.7 |  |  | 0.2 |  |  | 0.4 |
| Low (<2.79) | 9 | 27 |  | 12 | 24 |  | 03 | 10 | 23 |  | 4 | 32 |  | 2 | 34 |  | 3 | 33 |  |
| Medium (2.79-3.32) | 7 | 26 |  | 25 | 08 |  | 00 | 15 | 18 |  | 6 | 27 |  | 4 | 29 |  | 5 | 28 |  |
| High (>3.32) | 10 | 25 |  | 25 | 10 |  | 00 | 15 | 20 |  | 6 | 29 |  | 7 | 28 |  | 2 | 33 |  |
| **site 3** |  |  | 0.7 |  |  | 0.03 |  |  |  | 0.2 |  |  | 0.6 |  |  | 0.3 |  |  | 0.6 |
| Low (<1.50) | 7 | 27 |  | 26 | 8 |  | 1 | 16 | 17 |  | 4 | 30 |  | 3 | 31 |  | 4 | 30 |  |
| Medium (1.50-1.88) | 9 | 27 |  | 17 | 19 |  | 2 | 9 | 25 |  | 5 | 31 |  | 4 | 32 |  | 4 | 32 |  |
| High (>1.88) | 10 | 24 |  | 19 | 15 |  | 0 | 15 | 19 |  | 7 | 27 |  | 6 | 28 |  | 2 | 32 |  |
| **site 4** |  |  | 0.2 |  |  | 0.2 |  |  |  | 0.4 |  |  | 0.4 |  |  | 0.6 |  |  | 0.5 |
| Low (<2.14) | 9 | 26 |  | 25 | 10 |  | 1 | 13 | 21 |  | 6 | 29 |  | 3 | 32 |  | 5 | 30 |  |
| Medium (2.14-2.70) | 5 | 29 |  | 17 | 17 |  | 2 | 11 | 21 |  | 3 | 31 |  | 4 | 30 |  | 3 | 31 |  |
| High (>2.70) | 12 | 23 |  | 20 | 15 |  | 0 | 16 | 19 |  | 7 | 28 |  | 6 | 29 |  | 2 | 33 |  |
| **site 5** |  |  | 0.9 |  |  | 0.002 |  |  |  | 0.4 |  |  | 0.2 |  |  | 0.6 |  |  | 0.01 |
| Low (<1.59) | 9 | 28 |  | 14 | 23 |  | 2 | 13 | 22 |  | 3 | 34 |  | 3 | 34 |  | 5 | 32 |  |
| Medium (1.59-1.85) | 8 | 24 |  | 25 | 7 |  | 0 | 11 | 21 |  | 7 | 25 |  | 5 | 27 |  | 5 | 27 |  |
| High (>1.85) | 9 | 26 |  | 23 | 12 |  | 1 | 16 | 18 |  | 6 | 29 |  | 5 | 30 |  | 0 | 35 |  |
| **site 6** |  |  | 0.7 |  |  | 0.008 |  |  |  | 0.2 |  |  | 0.3 |  |  | 0.2 |  |  | 0.4 |
| Low (<1.65) | 7 | 28 |  | 28 | 07 |  | 1 | 17 | 17 |  | 5 | 30 |  | 2 | 33 |  | 3 | 32 |  |
| Medium (1.65-2.14) | 9 | 25 |  | 17 | 17 |  | 2 | 9 | 23 |  | 3 | 31 |  | 4 | 30 |  | 5 | 29 |  |
| High (>2.14) | 10 | 25 |  | 17 | 18 |  | 0 | 14 | 21 |  | 8 | 27 |  | 7 | 28 |  | 2 | 33 |  |
| **site 7** |  |  | 0.4 |  |  | 0.1 |  |  |  | 0.08 |  |  | 0.4 |  |  | 0.3 |  |  | 0.6 |
| Low (<3.09) | 9 | 25 |  | 25 | 9 |  | 3 | 13 | 18 |  | 3 | 31 |  | 3 | 31 |  | 4 | 30 |  |
| Medium (3.09-3.98) | 6 | 29 |  | 19 | 16 |  | 0 | 11 | 24 |  | 6 | 29 |  | 3 | 32 |  | 4 | 31 |  |
| High (>4.08) | 11 | 24 |  | 18 | 17 |  | 0 | 16 | 19 |  | 7 | 28 |  | 7 | 28 |  | 2 | 33 |  |
| **Mean (7 sites)** |  |  | 0.3 |  |  | 0.7 |  |  |  | 0.1 |  |  | 0.3 |  |  | 0.2 |  |  | 0.6 |
| Low (<2.15) | 8 | 30 |  | 21 | 17 |  | 3 | 13 | 22 |  | 4 | 34 |  | 2 | 36 |  | 4 | 34 |  |
| Medium (2.15-2.60) | 6 | 25 |  | 20 | 11 |  | 0 | 11 | 20 |  | 4 | 27 |  | 4 | 27 |  | 4 | 27 |  |
| High (>2.60) | 12 | 23 |  | 21 | 14 |  | 0 | 16 | 19 |  | 8 | 27 |  | 7 | 28 |  | 2 | 33 |  |
| **p-53** |  |  |  |  |  |  |  |  |  |  |  |  |  |  |  |  |  |  |  |
| **site 1** |  |  | 0.6 |  |  | 0.0001 |  |  |  | 0.5 |  |  | 0.06 |  |  | 0.03 |  |  | 0.02 |
| Low (<3.18) | 9 | 23 |  | 5 | 27 |  | 1 | 11 | 20 |  | 9 | 23 |  | 1 | 31 |  | 2 | 30 |  |
| Medium (3.18-3.74) | 8 | 24 |  | 23 | 9 |  | 2 | 10 | 20 |  | 2 | 30 |  | 3 | 29 |  | 1 | 31 |  |
| High (>3.74) | 6 | 27 |  | 30 | 3 |  | 0 | 14 | 19 |  | 5 | 28 |  | 8 | 25 |  | 8 | 25 |  |
| **site 2** |  |  | 0.2 |  |  | 0.0001 |  |  |  | 0.9 |  |  | 0.6 |  |  | 0.7 |  |  | 0.1 |
| Low (< 8.68) | 11 | 21 |  | 7 | 25 |  | 1 | 11 | 20 |  | 4 | 28 |  | 3 | 29 |  | 1 | 31 |  |
| Medium (8.68-11.60) | 7 | 25 |  | 19 | 13 |  | 1 | 10 | 21 |  | 7 | 25 |  | 5 | 27 |  | 6 | 26 |  |
| High (>11.60) | 5 | 28 |  | 32 | 1 |  | 1 | 14 | 18 |  | 5 | 28 |  | 4 | 29 |  | 4 | 29 |  |
| **site 3** |  |  | 0.04 |  |  | 0.0001 |  |  |  | 0.6 |  |  | 0.3 |  |  | 0.03 |  |  | 0.1 |
| Low (<3.34) | 10 | 22 |  | 4 | 28 |  | 3 | 11 | 27 |  | 7 | 25 |  | 1 | 31 |  | 1 | 31 |  |
| Medium (3.34-4.18) | 10 | 22 |  | 23 | 9 |  | 0 | 15 | 18 |  | 6 | 26 |  | 3 | 29 |  | 4 | 28 |  |
| High (>4.18) | 3 | 30 |  | 31 | 2 |  | 0 | 9 | 14 |  | 3 | 30 |  | 8 | 25 |  | 6 | 27 |  |
| **site 4** |  |  | 0.2 |  |  | 0.02 |  |  |  | 0.1 |  |  | 0.3 |  |  | 0.0001 |  |  | 0.06 |
| Low (<4.31) | 11 | 22 |  | 15 | 18 |  | 2 | 9 | 22 |  | 8 | 25 |  | 0 | 33 |  | 3 | 30 |  |
| Medium (4.31-5.46) | 7 | 24 |  | 17 | 14 |  | 1 | 9 | 21 |  | 3 | 28 |  | 1 | 30 |  | 1 | 30 |  |
| High (>5.46) | 5 | 28 |  | 26 | 7 |  | 0 | 17 | 16 |  | 5 | 28 |  | 11 | 22 |  | 7 | 26 |  |
| **Mean (4 sites)** |  |  | 0.04 |  |  | 0.0001 |  |  |  | 0.2 |  |  | 0.7 |  |  | 0.006 |  |  | 0.06 |
| Low (<5.16) | 10 | 23 |  | 3 | 30 |  | 1 | 8 | 24 |  | 7 | 26 |  | 1 | 32 |  | 1 | 32 |  |
| Medium (5.16-6.22) | 10 | 21 |  | 25 | 6 |  | 2 | 13 | 16 |  | 4 | 27 |  | 2 | 29 |  | 3 | 28 |  |
| High (>6.22) | 3 | 30 |  | 30 | 3 |  | 0 | 14 | 19 |  | 5 | 28 |  | 9 | 24 |  | 7 | 26 |  |
| **RUNX-3** |  |  |  |  |  |  |  |  |  |  |  |  |  |  |  |  |  |  |  |
| **site 1** |  |  | 0.003 |  |  | 0.06 |  |  |  | 0.2 |  |  | 0.8 |  |  | 0.08 |  |  | 0.3 |
| Low (<1.09) | 5 | 28 |  | 25 | 8 |  | 0 | 16 | 17 |  | 4 | 29 |  | 3 | 30 |  | 2 | 31 |  |
| Medium (1.09-1.85) | 15 | 19 |  | 17 | 17 |  | 2 | 9 | 23 |  | 6 | 28 |  | 2 | 32 |  | 3 | 31 |  |
| High (>1.85) | 4 | 30 |  | 18 | 16 |  | 1 | 12 | 21 |  | 6 | 28 |  | 8 | 26 |  | 6 | 28 |  |
| **site 2** |  |  | 0.6 |  |  | 0.09 |  |  |  | 0.5 |  |  | 0.9 |  |  | 0.03 |  |  | 0.5 |
| Low (<0.80) | 7 | 26 |  | 16 | 17 |  | 1 | 11 | 21 |  | 6 | 27 |  | 4 | 29 |  | 4 | 29 |  |
| Medium (0.80-1.56) | 7 | 27 |  | 19 | 15 |  | 2 | 12 | 20 |  | 5 | 29 |  | 1 | 33 |  | 2 | 32 |  |
| High (>1.56) | 10 | 24 |  | 25 | 9 |  | 0 | 14 | 20 |  | 5 | 29 |  | 8 | 26 |  | 5 | 29 |  |
| **site 3** |  |  | 0.1 |  |  | 0.3 |  |  |  | 0.9 |  |  | 0.001 |  |  | 0.3 |  |  | 0.4 |
| Low (<0.48) | 6 | 29 |  | 24 | 11 |  | 1 | 15 | 19 |  | 0 | 35 |  | 5 | 30 |  | 2 | 33 |  |
| Medium (0.48-1.06) | 12 | 20 |  | 19 | 13 |  | 1 | 10 | 21 |  | 8 | 24 |  | 2 | 30 |  | 4 | 28 |  |
| High (>1.06) | 6 | 28 |  | 17 | 17 |  | 1 | 12 | 21 |  | 8 | 26 |  | 6 | 28 |  | 5 | 29 |  |
| **Mean (3 sites)** |  |  | 0.4 |  |  | 0.2 |  |  |  | 0.3 |  |  | 0.6 |  |  | 0.3 |  |  | 0.3 |
| Low (<0.92) | 6 | 27 |  | 24 | 9 |  | 0 | 14 | 19 |  | 4 | 29 |  | 3 | 30 |  | 2 | 31 |  |
| Medium (0.92-1.43) | 11 | 23 |  | 18 | 16 |  | 2 | 9 | 23 |  | 5 | 29 |  | 3 | 31 |  | 3 | 31 |  |
| High (>1.43) | 7 | 27 |  | 18 | 16 |  | 1 | 14 | 19 |  | 7 | 27 |  | 7 | 27 |  | 6 | 28 |  |

# The numbers shown correspond to successful methylation analysis at each locus and may vary from on locus to the other.

* The cut off points for each locus were categorized based on control levels shown between parentheses as follows: low (< 33 percentile), medium (≥ 33 and < 66 percentile), and high (≥ 66% percentile).

*p*•Chi-squared p-value for comparison of low, medium, and high methylation levels between levels of factor.
